# Gibson assembly in human cells

**DOI:** 10.1016/j.omtn.2026.103151

**Published:** 2026-11-09

**Authors:** Sébastien Levesque, Daniel E. Bauer

**Affiliations:** 1 CHU de Québec Research Center – Université Laval, Québec, QC, Canada; 2 Department of Pediatrics, Faculty of Medicine, Université Laval, Québec, QC, Canada; 3 Division of Hematology/Oncology, Boston Children’s Hospital, Boston, MA, USA; 4 Department of Pediatric Oncology, Dana-Farber Cancer Institute, Boston, MA, USA; 5 Harvard Stem Cell Institute, Cambridge, MA, USA; 6 Broad Institute, Cambridge, MA, USA; 7 Department of Pediatrics, Harvard Medical School, Boston, MA, USA

## Main text

CRISPR-based technologies are transforming gene therapy for severe genetic disorders, but developing a personalized genome-editing approach for every pathogenic mutation seems unfeasible. Conversely, replacing a defective DNA sequence with a functional one could enable the development of universal, mutation-agnostic gene therapies that could be accessible to patients. Although promising, achieving medium- to large-size genetic modifications remains challenging in therapeutically relevant cells. In our recently published manuscript in *Nature*,^1^ we describe prime assembly, a genome-editing modality that enables targeted DNA sequence assembly and integration in human cells (Figure 1).

**Figure 1:**
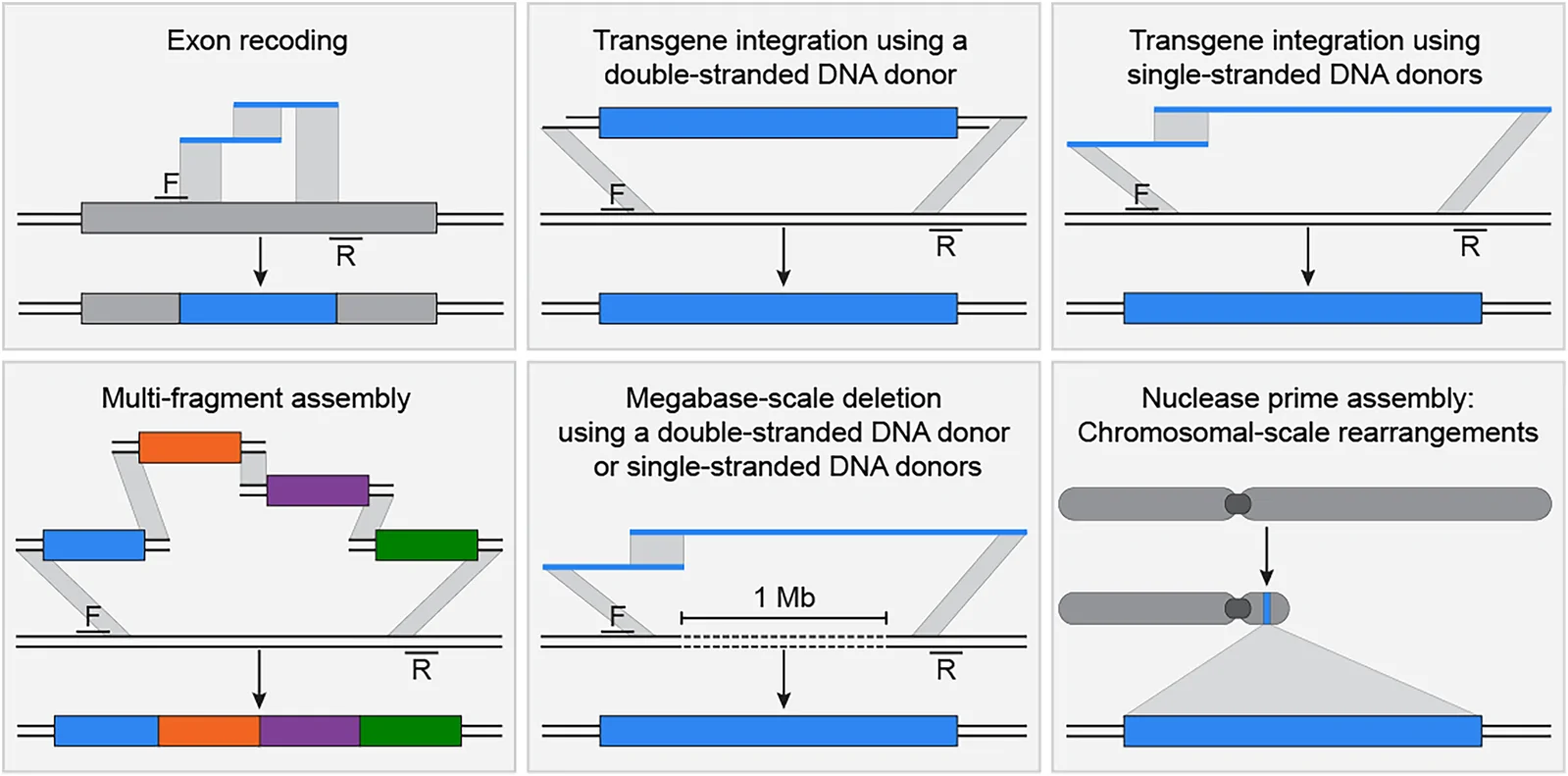
Versatility of prime assembly for genome engineering in human cells

Aiming to develop therapeutic genome-editing approaches for severe blood disorders using prime editing,^2^ a technology known for its high genome-wide specificity, we first designed an approach to recode an exon in *TINF2*, which harbors a hotspot of dominant gain-of-function mutations that cause short telomeres and a bone marrow failure syndrome known as dyskeratosis congenita. After optimization, we achieved efficient twin prime editing, but in 2023, these prime-editing guide RNAs (pegRNAs) were too long to be chemically synthesized for experiments in hematopoietic stem and progenitor cells (HSPCs). We then designed a strategy to bridge this gap by recoding the exon using single-stranded DNA (ssDNA) donors homologous to 3′ flaps generated by shorter pegRNAs. We were amazed to observe efficient targeted genomic integration using prime editing and DNA donors. Reminiscent of Gibson assembly cloning,^3^ which uses complementary 3′ single-stranded DNA overhangs, we named this approach prime assembly.

We then tested whether we could achieve targeted transgene integration using fluorescent reporters. We successfully integrated reporter transgenes at different genomic loci in three human cell lines using double-stranded DNA (dsDNA) donors. Importantly, prime assembly performed similarly to or better than other gene-targeting platforms under comparable conditions. We then developed a method named Donor-Seq to assess the genome-wide integration profile of prime assembly donors. Using Donor-Seq, we observed high genome-wide specificity with prime assembly, which compares favorably with previous Cas9 nuclease-based approaches that displayed frequent guide-dependent integration at off-target sites.

One advantage of Gibson assembly cloning is its ability to assemble multiple DNA fragments, and we reasoned that prime assembly could similarly enable multi-fragment assembly and integration in human cells. We split our DNA donors into two, three, or four fragments and achieved efficient multi-fragment prime assembly. We also tested the impact of increasing the distance between the two pegRNAs and efficiently replaced genomic sequences of one to ten kilobases with an *EGFP* reporter payload. Under puromycin selection pressure, we achieved targeted deletions of one million base pairs. Finally, using a nuclease prime editor, we also introduced targeted chromosome-scale rearrangements, including chromosome arm deletion, translocation, and megabase-scale inversion. For functional genomics applications, prime assembly could facilitate the assembly, integration, and screening of libraries directly in human cells when constrained by DNA synthesis, as well as the introduction of large-scale structural rearrangements to haploidize specific genomic loci or model diseases.

One key challenge for targeted transgene integration in primary hematopoietic cells is the toxicity associated with dsDNA donors. We thus adapted our approach and achieved targeted *EGFP* and *PuroR* integration and expression at diverse loci using ssDNA donors, albeit at lower efficiency than with dsDNA donors. Importantly, we successfully integrated a transgene at three therapeutically relevant loci in primary CD34^+^ HSPCs and CD3^+^ T cells from multiple healthy donors. Using a CDK4/6 inhibitor in K562 cells or a low concentration of cytokines to maintain primary CD3^+^ T cell in a resting state, we confirmed that prime assembly remained active in non-dividing cells.

In May 2025, we presented our prime assembly study at the Annual Meeting of the American Society of Gene & Cell Therapy in New Orleans. After the conference, colleagues at the University of Massachusetts generously communicated unpublished results regarding their independently developed Gibson-like genome editing modality. We agreed to use the same name (prime assembly), share our manuscripts as preprints on the *bioRxiv* server, and co-submit them to *Nature*. We continued to share updates throughout the revision process, and our revised manuscripts have now been published.^1^^,^^4^ Although apparently mechanistically distinct, other prime editing-based gene targeting modalities that depend on plasmid dsDNA donors have also been recently published.^5^^,^^6^ Collectively, our studies support the use of prime editors to install medium- to large-scale genomic modifications without relying on recombinases.^1^^,^^4^^,^^5^^,^^6^

Our studies also provide blueprints to better understand and improve prime assembly in human cells. Notably, we observed a marked increase in efficiency and product purity when inhibiting competing non-homologous end joining (NHEJ) and microhomology-mediated end joining (MMEJ) pathways. Prime assembly is associated with precise and imprecise outcomes, mainly flap integration with or without deletion events. Our initial CRISPResso2 parameters failed to capture some of the imprecise events, and during revision, we developed allele classification pipelines to precisely quantify the different types of prime assembly events using amplicon sequencing. We also designed droplet-digital PCR (ddPCR) drop-off assays to broadly quantify WT allele disruption events when performing targeted transgene integration. Although prime assembly offers high genome-wide specificity, it can also generate frequent imprecise on-target events without optimization, and we recommend using complementary approaches to characterize and quantify these diverse allelic outcomes.

We also observed that the optimal concentration of ssDNA donors in the nucleofection mix ranged from 800–1,600 nM, while less than 200 nM of long ssDNA or dsDNA donors could be used without overt toxicity. The local concentration of donor available at the prime assembly site may be limiting, and future efforts at recruiting donors to their complementary 3′ flaps could enhance DNA assembly and integration.

A new paradigm emerged in 2009 when Gibson and colleagues demonstrated the *in vitro* assembly of kilobase DNA molecules.^3^ Although it never gained widespread adoption, molecular cloning through *in cellulo* DNA assembly in *Escherichia coli* was reported in the 1990s.^7^^,^^8^^,^^9^ More than three decades later, we demonstrate that human cells also support *in cellulo* DNA assembly, allowing targeted genomic integration and rearrangements.^1^^,^^4^ Overall, prime assembly expands the genome-editing toolbox by enabling RNA-programmable exon recoding, transgene integration, and megabase-scale rearrangements without relying on cell-cycle progression, dsDNA donors, or nuclease-driven double-strand breaks. Elucidating the molecular mechanisms and DNA repair factors involved in prime assembly could further improve its efficiency and precision, opening opportunities for functional genomics and mutation-agnostic gene therapy.

## Acknowledgments

S.L. was supported by the Foundation of the CHU de Québec, a Discovery Grant (RGPIN-2026-07315) from the Natural Sciences and Engineering Research Council of Canada, a Next Generation of Scientists Award (Grant 26097) from the Cancer Research Society, and a BMO Excellence Grant from the Pediatric Research Foundation. D.E.B. was supported by the Doris Duke Foundation (2022092), the St. Jude Children’s Research Hospital Collaborative Research Consortium, the Harvard Stem Cell Institute and the National Institutes of Health (R01HG013618 and R01HL165061).

## Declaration of interests

S.L. and D.E.B. have filed a patent application (WO 2025/038842 A1) covering prime assembly technology.
